# Machine Learning-Based Differentiation of Nontuberculous Mycobacteria Lung Disease and Pulmonary Tuberculosis Using CT Images

**DOI:** 10.1155/2020/6287545

**Published:** 2020-09-29

**Authors:** Zhiheng Xing, Wenlong Ding, Shuo Zhang, Lingshan Zhong, Li Wang, Jigang Wang, Kai Wang, Yi Xie, Xinqian Zhao, Nan Li, Zhaoxiang Ye

**Affiliations:** ^1^Tianjin Medical University Cancer Institute and Hospital, National Clinical Research Center for Cancer, Key Laboratory of Cancer Prevention and Therapy, Tianjin, Tianjin's Clinical Research Center for Cancer, Tianjin, China; ^2^Haihe Hospital, Tianjin University, Tianjin Institute of Respiratory Diseases, Tianjin, China

## Abstract

An increasing number of patients infected with nontuberculous mycobacteria (NTM) are observed worldwide. However, it is challenging to identify NTM lung diseases from pulmonary tuberculosis (PTB) due to considerable overlap in classic manifestations and clinical and radiographic characteristics. This study quantifies both cavitary and bronchiectasis regions in CT images and explores a machine learning approach for the differentiation of NTM lung diseases and PTB. It involves 116 patients and 103 quantitative features. After the selection of informative features, a linear support vector machine performs disease classification, and simultaneously, discriminative features are recognized. Experimental results indicate that bronchiectasis is relatively more informative, and two features are figured out due to promising prediction performance (area under the curve, 0.84 ± 0.06; accuracy, 0.85 ± 0.06; sensitivity, 0.88 ± 0.07; and specificity, 0.80 ± 0.12). This study provides insight into machine learning-based identification of NTM lung diseases from PTB, and more importantly, it makes early and quick diagnosis of NTM lung diseases possible that can facilitate lung disease management and treatment planning.

## 1. Introduction

Nontuberculous mycobacteria (NTM) is a major cause of morbidity and mortality in progressive lung diseases; unfortunately, an increasing number of patients with NTM lung disease (NTM-LD) are witnessed worldwide [1, 2]. As the etiologic agents, NTM have been found in a variety of environmental sources, and the clinical relevance of NTM-LD indicates the geographical heterogeneity in distribution and pathogenicity [3, 4]. Due to similar manifestations, it is difficult to recognize the lung infection caused by NTM or by pulmonary tuberculosis (PTB) for early diagnosis [5–9]. In clinic, as the first choice, microscopic examination of sputum smear for acid-fast bacillus (AFB) is used to screen mycobacterial lung infections; however, the presence of pulmonary mycobacterial infection could also be traced by AFB-positive [10–13]. Besides elaborate safety precautions, a definite diagnosis of NTM based on bacterial culture and strain identification lasts for about two months each time [6, 14]. Once being suspected of PTB with positive sputum AFB, a patient will take empirical anti-TB medicine for treatment when the test is ongoing to identify the bacteria. That means a part of patients receive potentially unnecessary treatment. It might cause the patients the risk of drug adverse reaction and thus nonessential healthcare cost [14]. Therefore, early diagnosis of NTM-LD can improve patients' life quality and facilitate disease treatment, and in particular, it benefits developing countries with resource-poor healthcare systems [1–3].

One challenging task is to differentiate NTM-LD from PTB lung disease (PTB-LD). Clinical manifestations are first considered, such as chronic cough, sputum production, and appetite loss. Moreover, clinical and radiographic characteristics are investigated, such as age, history of smoking, and previous TB treatment, since these characteristics are more frequently found in patients with NTM-LD than those with PTB-LD. However, considerable overlaps exist in classic manifestations, clinical characteristics, and radiographic features, making the diagnosis subjective and unstable [7–10, 14–19]. According to the radiographic features of cavities and bronchiectasis, NTM-LD can be generally classified into two distinct subtypes. One is characterized by cavities with areas of increased opacity and usually located in the upper lobes, and the other is by bronchiectasis and bronchiolar nodules which are predominant in the middle lobe and/or lingual. In comparison to PTB-LD patients with cavities or bronchiectasis, CT findings indicate that radiographic changes of NTM-LD could lead to subtle differences, such as thin-walled cavities and less bronchogenic but more contiguous spread of disease [14, 16, 17]. However, these observed differences are qualitative or subtle, which are not sufficient or discriminative to differ the NTM-LD from PTB-LD patients.

Some studies have explored machine learning methods for PTB screening. An artificial neural network (ANN) was used for the prediction of PTB infection [20]. The study examined blood samples of 115 PTB-LD patients and 60 normal subjects. Based on 39 features, the accuracy of two-hidden-layered ANN was up to 93.93%. An approach incorporating a fuzzy logic controller and an artificial immune recognition system was proposed [21] which utilized 20 features to represent each of 175 data samples and resulted in high accuracy, sensitivity, and specificity. A convolutional neural network (CNN) was designed for PTB examination [22]. The network enabled an end-to-end training from images to labels and required no objective-specific manual feature engineering. Its classification performance was larger than 0.85 (AUC (area under the curve)) on three real data sets [22]. Transferred learning, deep network, data augmentation, and radiologist involvement were considered, and high performance of PTB diagnosis was achieved [23]. These machine learning approaches are advancing the techniques for PTB-LD diagnosis [24].

The present study explores to build a machine learning model for the differentiation of NTM-LD and PTB-LD by using CT images. To the best of our knowledge, there are no machine learning models available to this challenging task. The contribution of this study is manifold. First, a machine learning approach is designed. It involves 116 patients, and to each patient case, 103 quantitative features are analyzed. Second, the effectiveness of different regions (cavities, bronchiectasis, and their combination) is investigated. Third, experimental results indicate that bronchiectasis is more informative, and two discriminative features are figured out. In addition, a simple and interpretable machine learning model is built which achieves promising classification performance. This study provides insight into machine learning-based differentiation of NTM-LD and PTB-LD patients, and most importantly, it provides some feasible clues on the early and quick diagnosis of lung diseases, benefiting disease management and treatment planning.

## 2. Material and Methods

### 2.1. Data Collection

From January 2019 to January 2020, a total of 1291 AFB smear-positive sputum specimens of previously untreated cases were retrospectively retrieved in Tianjin Haihe Hospital, Tianjin University, China. The sputum test is required to be conducted at least twice to show varying degrees of AFB smear positive. After being cultured and strain-identified, the smear-positive sputum was tested. The test result verified that 287 specimens were NTM, and 1004 were PTB. Details of PTB and NTM diagnosis are as follows. In order to find the mycobacteria in a tissue section, an AFB stain is done for all sputum samples. Based on PCR assays, a TB polymerase chain reaction (PCR) was performed with in-house IS6110. Mycobacterium culture was carried out using Löwenstein-Jensen Medium. Specifically, PTB diagnosis was in accordance with mycobacteria culture results and guidelines from the Chinese Medical Association, and NTM was based on mycobacterial culture results and guidelines of the American Thoracic Society (ATS) [25].

**Figure 1:**
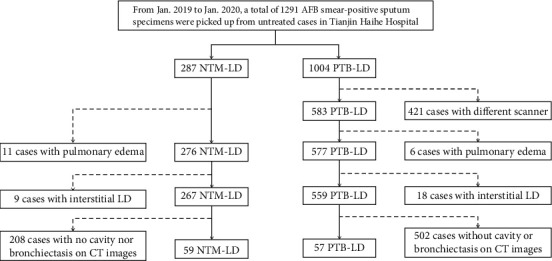
The procedure of data collection. After review of CT images, 116 cases remain for analysis.

The chosen patients were with reliable CT imaging data, and CT scan images were reviewed independently by three experienced radiologists (XZH, WL, and ZS) who were blind to patients' microbiology results. With regard to the chest CT findings, the final decisions were determined by consensus. As shown in Figure 1, after an independent review of CT images, 116 cases (57 M. tuberculosis and 59 NTM) with lung cavities and/or with bronchiectasis were identified for retrospective analysis.

In addition, clinical characteristics of patients in both groups are shown in Table 1. It indicates that most patients show similar symptoms, including cough, sputum production, and fever. It is also found that some patients are smokers and some are with diabetes mellitus. Most importantly, no significant difference in symptoms is found between the two groups of patients.

### 2.2. CT Image Acquisition

All chest CT examinations were performed within 3 months of the AFB smear test by using a helical CT scanner (Aquilion Prime 128, Canon Medical Systems, Otawara, Japan). Patients were scanned from the lung apices to the adrenal glands during full inspiration, and the procedure was repeated during full expiration. The CT scanning parameters were as follows: 64 × 0.5 mm collimation, 120 kV automatic tube current modulation, and 0.5 s gantry rotation time. Contiguous inspiratory CT images were obtained with a thickness of 5.0 mm, at 5.0 mm intervals. Images were exported in DICOM format and forwarded to observers. In addition, CT scans were interpreted at window settings that were optimal for lung parenchyma (reconstruction kernel, FC 52; window level, -600 HU; window width, 1500 HU) and soft tissue (reconstruction kernel, FC 30; window level, 400 HU; window width, 40 HU).

### 2.3. Label Annotation

**Figure 2:**
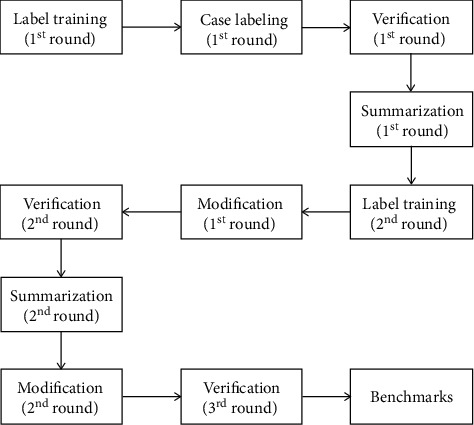
The procedure of cavitary and bronchiectasis annotation. Seven radiologists participated in this task. Six radiologists were trained in a trial-and-error manner (training, labeling, and modification), and one senior radiologist helped the verification, summarization, and training of the six radiologists.

Both cavitary and bronchiectasis are labeled by using the software 3D Slicer (version 3.10.2, http://www.slicer.org/). Seven radiologists participated in this task. To ensure the accuracy, six radiologists (1 to 3 years' experience) were trained in a trial-and-error manner. Furthermore, to ensure the consistency, after training and case annotation, a senior radiologist with 10 years' experience performed the label verification without clinical information. Meanwhile, the senior radiologist performed as a supervisor and summarized the errors and cautions in label annotation and further gave the junior radiologists a second chance to rectify their errors. As shown in Figure 2, the whole procedure involves 2-round training, 2-round case labeling, 2-round modification, 2-round summarization, and 3-round verification until the labels can be used for the follow-up analysis.

**Figure 3:**
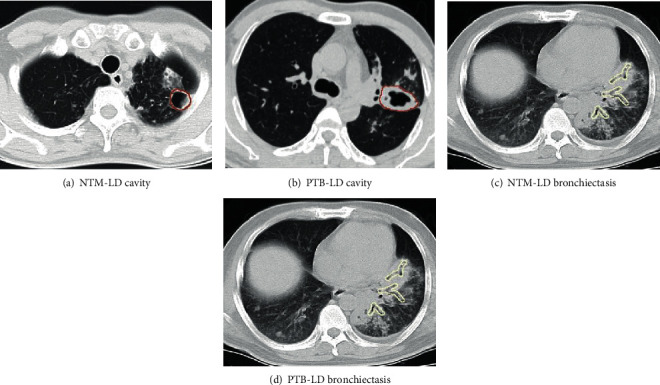
Representative examples of annotated cavity and bronchiectasis. Thick-walled, thin-walled, and wall-less cavities are marked as a cavity, and the outer wall of the lesion edge is the boundary mark, while bronchiectasis annotation should concern bronchial dilatation with respect to different factors.

Figure 3 shows representative examples of cavity (red) and bronchiectasis (yellow) from NTM-LD and PTB-LD patients. In CT images, both cavity and bronchiectasis are well-defined [26]. A cavity is a gas-filled space which is seen as a lucency or low-attenuation area, within pulmonary consolidation, a mass, or a nodule, and notably, no content is in a cavity. A thin-walled purification cavity is with a basically uniform wall thickness less than 3 mm and a thick-walled purification cavity is with a substantially uniform wall thickness greater than or equal to 3 mm, while a wall-less cavity is a gas density stove with no walls and smooth inner edges and located in the consolidated lung tissue. In addition, cavitary is a cavity that can be clearly imaged on the basis of consolidation. Whether a thick or thin wall, it is always marked as a cavity, and the outer wall of the lesion edge is the boundary mark. Morphological criteria of bronchiectasis consider bronchial dilatation with respect to accompanying pulmonary artery (signet ring sign), lack of tapering of bronchi, and identification of bronchi within 1 cm of the pleural surface. There are three types of labeling for bronchiectasis: (1) saccular: the inner diameter of the bronchus greater than 1.5 times the diameter of the accompanying artery. (2) Columnar: dilated bronchi with the same proximal and distal ends of the bronchi, longer than 2 cm. (3) Varicose veins: dilated bronchus with an uneven wall and tortuous course. The inner wall was marked as the boundary.

### 2.4. Feature Extraction

The open-source package Pyradiomics (https://pyradiomics.readthedocs.io) was used in this study, and 103 features were extracted regarding annotated bronchiectasis and cavity in original-resolution CT images. The features consist of 14 shape features, 21 first-order features, 22 Gray-Level Cooccurrence Matrix (GLCM) features, 16 Gray-Level Run Length Matrix (GLRLM) features, 16 Gray-Level Size Zone Matrix (GLSZM) features, and 14 Gray-Level Differential Matrix (GLDM) features. These features have been widely used for data representation and disease diagnosis [27, 28].

### 2.5. A Machine Learning Approach

A simple and interpretable machine learning approach is desirable. Given the data, to simplify the retrieval of informative features, Gini importance is used to measure the feature importance, since it defines dependence and independence of variables [29]. Further, to reduce the computation burden, several important features are considered in the follow-up analysis. Due to limited patient cases, to retrieve a few discriminative features is reasonable. At last, for good interpretability, linear SVM [30] performs the differentiation of the NTM-LD and the PTB-LD patients.

**Figure 4:**
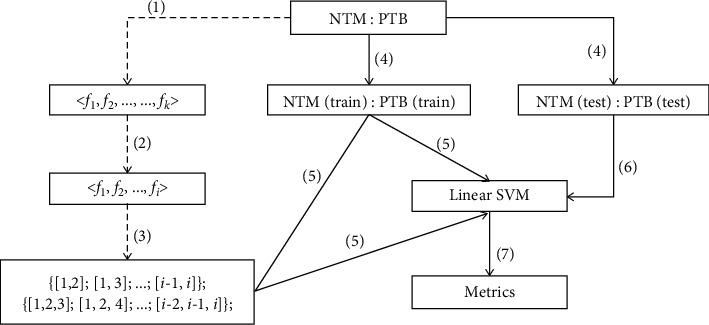
The framework for machine learning-based differentiation of NTM-LD and PTB-LD patients. The dashed lines indicate offline processing, and the solid ones stand for the retrieval of discriminative features for accurate disease diagnosis.

Figure 4 shows the flow chart which attempts to build a machine learning approach for interpretable diagnosis. The dashed lines indicate offline feature ranking. Features are sorted in terms of Gini importance. Assuming *k* features are extracted from each data sample, a resultant vector <*f*_1_, *f*_2_, ⋯, *f*_*k*_> stands for the indexes of the most to the least important features (1). Then, *i* top most important features are kept (2), and all combinations of feature subsets using 2 or 3 features are provided (3).

Potential feature subsets are prepared, and the optimal one is selected by comparing classification performance as shown in solid lines in Figure 4. For instance, if a subset of features is selected, the patient cases were randomly grouped into the training and the testing set (4). Using the training set, the parameters of the linear SVM classifier are experimentally determined (5). Once the model is trained, the testing set is fed into the model (6), and the performance is evaluated with classification metrics (7).

### 2.6. Experiment Design

Four experiments are conducted, and three are shown in Table 2. For each experiment, the number of patient cases, sex, and ages are reported. The first (TA), the second (TB), and the third (TC), respectively, use the cavity, the bronchiectasis, and both for retrieving the most discriminative features in an automated fashion. It should be noted that the fourth experiment is used to verify the effectiveness of the combination of retrieved features from TA and TB for disease classification.

With regard to each experiment, a total of 100 times of data splitting are conducted at random, and nearly 80% of cases are portioned into the training set and the rest into the testing set. After each time of data splitting, all feature subsets are used one by one for machine learning-based disease classification.

### 2.7. Performance Evaluation and Statistical Analysis

Four metrics are used to evaluate the classification performance, and they are the area under the curve (AUC), accuracy (ACC), sensitivity (SEN), and specificity (SPE). To figure out the best performance, i.e., the subset with the most discriminative features, statistical analyses were conducted using SPSS 17.0 software for Windows (SPSS Inc., Chicago, IL, USA), and performance metrics were compared by a paired *t*-test.

## 3. Results

### 3.1. Gini Importance-Based Feature Importance Ranking

Table 3 lists the top 10 most important features with regard to different forms used for lung disease analysis. The indexes of features that are derived from intensity statistics, shape representation, and texture analysis are, respectively, highlighted in italic, bold, and underline. Analysis of the cavitary form identifies 6 intensity statistics features and 4 texture analysis features, and analysis of the bronchiectatic form figures out 4 shape representation features and 6 texture analysis features, while analysis of the combined form indicates that all features are from the bronchiectatic form (feature indexes larger than 103), including one intensity statistics feature, three shape representation features, and six texture analysis features.

### 3.2. Cavity-Based Lung Disease Differentiation

Based on the cavity analysis and automated retrieval of discriminative features, three subsets achieving superior performance are listed in Table 4. It shows that the subset using the 22^nd^ and the 99^th^ features (in bold) obtains the best or competitive result in terms of four metrics, while no significant difference is found (*p* value > 0.23). The 30^th^ feature is also recognized as important; however, no improvement is observed in disease classification. As to the discriminative features, one (the 22^nd^) quantifies the intensity distribution, and the other (the 99^th^) shows the texture analysis of the cavity.

### 3.3. Bronchiectasis-Based Lung Disease Differentiation

Table 5 shows three subsets of features that lead to superior performance with regard to analyzing bronchiectasis. It suggests that the subset consisting of the 13^th^ and the 87^th^ features results in the best performance in terms of AUC and SPE, and the competitive performance in terms of ACC and SEN. It is worth noting that there is no significant difference of each performance metric between any two feature subsets (*p* value > 0.37). Moreover, the 48^th^ and the 6^th^ features are identified for their importance in disease differentiation, and adding one of them causes no enhancement. In the subset of discriminative features, one (the 13^th^) aims for shape representation, and the other (the 87^th^) analyzes tissue textures.

### 3.4. Combined Form for Lung Disease Differentiation

Based on both the cavity and the bronchiectasis, the subsets of features with good performance are presented in Table 6. The subset including the 190^th^ and the 152^nd^ features leads to the overall best performance in terms of three metrics (AUC, ACC, and SEN), and no significant difference is observed between the performance derived from each of the three subsets (*p* value > 0.52). Moreover, the 151^st^ feature is figured out for its importance in disease classification, while again, no improvement is found. In addition, both discriminative features are from texture analysis.

### 3.5. Performance Comparison

Table 7 shows the performance of lung disease differentiation with regard to different regions (TA: cavity; TB: bronchiectasis; TC: combined analysis by using automated feature selection; TD: combined analysis by using retrieved features from TA and TB). It demonstrates that the subset of retrieved features from the bronchiectasis (TB) is the most discriminative in comparison to each of the other retrieved features. It also indicates that combining feature subsets (TD) does not improve the differentiation performance, and on the contrary, a slight decrease is observed from each metric. In particular, it is found that the subset of features retrieved from the cavity results in inferior performance with AUC 0.70 on average.

**Figure 5:**
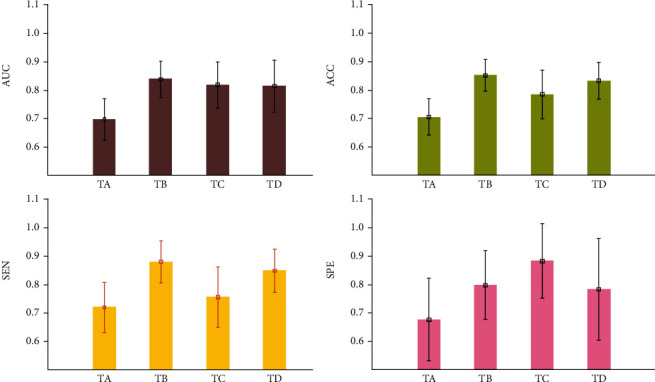
The performance of disease differentiation via analyzing different regions (TA, cavity; TB, bronchiectasis; TC, combined analysis using automated feature selection; TD, combined analysis using retrieved features from TA and TB). It shows that using bronchiectasis (TB) achieves overall best performance.

Error-bar plots in Figure 5 show the performance of lung disease differentiation by analyzing different regions. In general, using bronchiectasis (TB) achieves the highest AUC, ACC, and SEN and the second best SPE; using combined subsets of features (TD) obtains comparative performance, while using the cavity (TA) produces the worst performance in lung disease differentiation.

**Figure 6:**
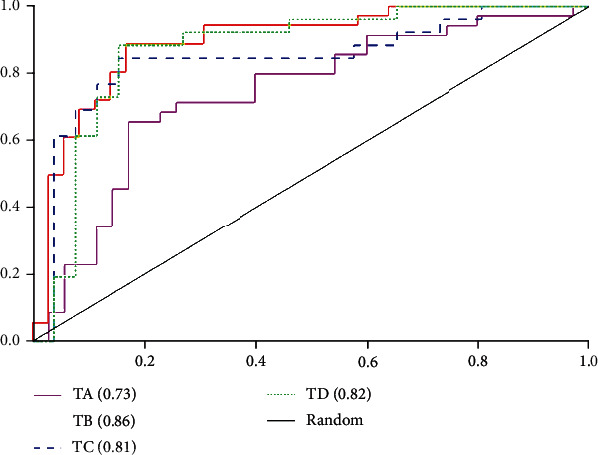
ROC curves of disease differentiation via the analysis of different regions.

ROC curves are shown in Figure 6. Different colors correspond to different methods. The bronchiectasis (TB, red) results in the best performance (AUC 0.86), followed by both regions with combined features (TD, green) with AUC 0.82 and both regions using automated feature selection (TC, blue) with AUC 0.81, and the worst is the cavitary form (TA, pink) with AUC 0.73.

## 4. Discussion

The increasing prevalence of NTM-LD is observed worldwide. Bacterial culture and strain identification remain the unique way to identify NTM, while the procedure takes a long time. Early and quick diagnosis of NTM-LD is urgently important yet challenging. Massive studies investigate the manifestations, clinical characteristics, radiographic findings, and clinical relevance. However, due to considerable overlap of symptoms and subtle difference in CT images, these findings are not sufficient to differentiate NTM-LD from PTB-LD patient cases. This study is the first work that explores machine learning to identify the NTM-LD patients from the PTB-LD ones, and in CT images, both the cavity and the bronchiectasis regions are delineated for quantitative analysis. Experimental results suggest that the proposed machine learning model achieves promising performance when two features are used to represent the bronchiectasis.

Quantified bronchiectasis plays an important role in the machine learning model for the differentiation between NTM-LD and PTB-LD cases. It enables high performance (AUC, 0.84 ± 0.06; ACC, 0.85 ± 0.06; SEN, 0.88 ± 0.07; and SPE, 0.80 ± 0.12) which is obviously higher than those corresponding metrics from the quantified cavity (AUC, 0.70 ± 0.07; ACC, 0.71 ± 0.06; SEN, 0.72 ± 0.09; and SPE, 0.68 ± 0.14). Its performance is slightly superior or competitive to that using both cavity and nodular bronchiectasis. Predominance of cavities and bronchiectasis is observed in radiographic findings of NTM-LD cases. One study indicated that of the 19 patients evaluated, 84.2% cases were with bronchiectasis, and 73.7% were with cavities [31]. One study with 34 patients figured out that nodular lesions (100%) and bronchiectasis (85.29%) were the most frequent CT features of Mycobacterium simiae pulmonary infection [32]. A meta-analysis study reported that 9.3% of NTM-LD patients were with bronchiectasis [33]. A comparison of CT findings between NTM-LD and PTB-LD has also been considered. A study analyzed 95 CT scans from 159 patients with AFB smear-positive sputum (75 scans from PTB-LD patients and 20 scans from NTM-LD patients) and claimed that the presence of bronchiectasis changes in CT scans was strongly associated with patients with NTM-LD [16]. A study investigated a total of 4167 untreated cases with AFB smear-positive sputum (124 cases were with NTM-LD, and 210 cases with PTB-LD were randomly selected from the remaining cases), and bronchiectasis and thin-walled cavity were identified independent predictors for NTM-LD diagnosis via multivariate analysis [14]. A cavity analysis study (128 NTM-LD and 128 PTB-LD patients with matched age and gender) discovered that the major cavities in NTM disease generally have thinner and more even walls than those in PTB cases [17]. Thus, to investigate cavity and bronchiectasis in CT images for lung disease differentiation is reasonable. Most importantly, the current study points out that the quantified bronchiectasis seems more informative than the cavity in differing the NTM-LD from PTB-LD cases.

The machine learning model is well built, and it is simple and interpretable. It makes use of two quantitative features for the representation of bronchiectasis in CT images. In the original images, one feature describes the minor (second-largest) axis length of shape, and the other is the zone entropy of GLSZM texture which describes the randomness in the distribution of zone sizes and gray levels. Interestingly, both features have been reported in related clinical studies. For instance, the minor axis length of shape is important in the detection of clinically significant prostate cancer in multiparametric MR images [34], and the zone entropy of GLSZM reflects the areas with different gray intensities within the nodules for lung cancer detection [35]. However, it should be noted that both features cannot be perceived directly, and thus, accurate segmentation of the bronchiectasis regions becomes indispensable. Moreover, the model utilizes an interpretable classifier of linear SVM, which is widely used in knowledge discovery. It is worth noting that SVM with a nonlinear kernel could map data samples into high-dimension space, and the classification performance might be further improved. In addition, this simple model supports good generalization and evolving, and it can avoid the curse of dimensionality in high-throughput feature analysis.

There are several limitations to the current study. First, the number of patient cases should be increased, and a multi-institution study would be better, as it can make the results more convincing, generalizable, and applicable. Therefore, our future work will focus on data collection and multicenter collaboration. Second, advanced techniques [23, 24, 27, 28] could be used to improve the diagnosis performance, and the hybrid techniques [36–38] that integrate manifestations and clinical and radiographic features are feasible. Third, automated annotation and quantification of bronchiectasis and cavity are also appealing. For instance, the thickness of cavity walls is helpful, since cavity walls of NTM-LD patients are found significantly thinner and more even than those of PTB-LD [17]. However, it requires advanced algorithms for accurate and objective quantification. In the end, this study involves a single hospital and a limited number of cases. For further verification of our findings, a large-scale experiment should be conducted.

## 5. Conclusion

The increasing incidence and prevalence of NTM-LD have become a major public health problem. This study explores a machine learning approach, and both bronchiectasis and cavity are delineated for differing NTM-LD patients from PTB-LD patients. Bronchiectasis is found more informative, and two quantitative features are identified discriminative for disease differentiation. The built machine learning model makes early and quick diagnosis of NTM-LD possible, and it could further facilitate disease management and treatment planning and improve patients' life quality.

## Figures and Tables

**Table 1:** Clinical characteristics of patients.

	NTM-LD (*n* = 59)	PTB-LD (*n* = 57)	Chi-squared test	*p* value
Cough	27 (45.76%)	36 (63.16%)	3.535	0.060
Sputum production	25 (42.37%)	31 (54.39%)	1.676	0.196
Fever	17 (28.81%)	20 (35.09%)	0.525	0.469
Chest pain	3 (5.08%)	8 (14.04%)	2.706	0.100
Hemoptysis	7 (11.86%)	7 (12.28%)	0.005	0.945
Fatigue	4 (6.78%)	1 (1.75%)	0.766	0.382
Emaciation	4 (6.78%)	2 (3.51%)	0.141	0.707
Shortness of breath	1 (1.69%)	4 (7.02%)	0.910	0.340
Smoker	15 (25.42%)	14 (24.56%)	0.011	0.915
Diabetes mellitus	9 (15.25%)	8 (14.04%)	0.034	0.853
COPD	5 (8.47%)	5 (8.77%)	0.000	1.000

COPD stands for chronic obstructive pulmonary disease; *p* < 0.05 indicates significant difference.

**Table 2:** The number of patient cases, sex, and age in experiment design.

	NTM (male/female/age)	PTB (male/female/age)
TA	44 (28/16/60 ± 15)	54 (40/14/48 ± 18)
TB	45 (28/17/62 ± 15)	54 (41/13/49 ± 17)
TC (TA∩TB)	32 (21/11/64 ± 12)	46 (34/12/49 ± 18)

**Table 3:** Ten most important features via Gini importance-based feature ranking.

Form	Ranked index of features from the most to less important ones									
Cavitary form	*2*	*23*	80	*35*	95	60	99	*22*	*30*	*25*
Bronchiectatic form	**13**	49	58	94	87	**7**	48	**11**	67	**6**
Combined form	*123*	190	**116**	152	**109**	161	197	170	**114**	151

**Table 4:** Cavity-based LD differentiation.

Feature subsets	AUC	ACC	SEN	SPE
**[99, 22]**	0.70 ± 0.07	0.71 ± 0.06	0.72 ± 0.09	0.68 ± 0.14
[99, 30]	0.70 ± 0.08	0.70 ± 0.08	0.70 ± 0.10	0.66 ± 0.15
[22, 99, 30]	0.69 ± 0.07	0.70 ± 0.07	0.72 ± 0.09	0.68 ± 0.11

^#^The 22^nd^ feature, original_firstorder_interquartilerange; the 30^th^ feature, original_firstorder_robustmeanabsolutedeviation; the 99^th^ feature, original_gldm_largedependencelowgraylevelemphasis.

**Table 5:** Bronchiectatic form-based differentiation of lung diseases.

Feature subsets	AUC	ACC	SEN	SPE
**[13, 87]**	0.84 ± 0.06	0.85 ± 0.06	0.88 ± 0.07	0.80 ± 0.12
[13, 87, 48]	0.82 ± 0.07	0.84 ± 0.07	0.89 ± 0.09	0.74 ± 0.13
[13, 87, 6]	0.83 ± 0.07	0.85 ± 0.07	0.89 ± 0.09	0.76 ± 0.10

^#^The 6^th^ feature, original_shape_leastaxislength; the 13^th^ feature, original_shape_minoraxislength; the 48^th^ feature, original_glcm_Imc1; the 87^th^ feature, original_glszm_zoneentropy.

**Table 6:** Disease differentiation using both the cavity and the bronchiectasis.

Feature subsets	AUC	ACC	SEN	SPE
**[190, 152]**	0.82 ± 0.08	0.78 ± 0.08	0.76 ± 0.11	0.88 ± 0.13
[190, 116, 152]	0.81 ± 0.10	0.75 ± 0.09	0.75 ± 0.06	0.89 ± 0.16
[190, 116, 151]	0.82 ± 0.10	0.77 ± 0.06	0.75 ± 0.06	0.86 ± 0.15

^#^The 116^th^ feature, original_shape_minoraxislength; the 151^st^ feature, original_glcm_Imc1; the 152^nd^ feature, original_glcm_Imc2; the 190^th^ feature, original_glszm_zoneentropy.

**Table 7:** LD differentiation using selected features with regard to different regions.

	Retrieved features	AUC	ACC	SEN	SPE
TA	[99, 22]	0.70 ± 0.07	0.71 ± 0.06	0.72 ± 0.09	0.68 ± 0.14
TB	**[13, 87]**	0.84 ± 0.06	0.85 ± 0.06	0.88 ± 0.07	0.80 ± 0.12
TC	[190, 152]	0.82 ± 0.08	0.78 ± 0.08	0.76 ± 0.11	0.88 ± 0.13
TD	[99, 22]+[13, 87]	0.81 ± 0.09	0.83 ± 0.07	0.85 ± 0.08	0.78 ± 0.18

## Data Availability

The CT images supporting the findings of this study are restricted by the Medical Ethics Committee of Haihe Hospital in order to protect patient privacy. If interested, requests for access to the extracted features can be made to the corresponding author Zhaoxiang Ye (yezhaoxiang@163.com).
